# Evaluating larval mosquito resource partitioning in western Kenya using stable isotopes of carbon and nitrogen

**DOI:** 10.1186/1756-3305-6-353

**Published:** 2013-12-12

**Authors:** Thomas M Gilbreath, Eliningaya J Kweka, Yaw A Afrane, Andrew K Githeko, Guiyun Yan

**Affiliations:** 1 Ecology and Evolutionary Biology, University of California, 3501b Hewitt Hall, Irvine, CA 92697, USA; 2 Division of Livestock and Human Diseases Vector Control, Mosquito Section, Tropical Pesticides Research Institute, Ngaramtoni, Off Nairobi Road, P.O. Box 3024, Arusha, Tanzania; 3 Centre for Global Health Research, Kenya Medical Research Institute, P. O. Box 1578, Kisumu 40100, Kenya; 4 Program in Public Health, University of California, Irvine, CA 92697, USA

**Keywords:** *An. gambiae*, Stable isotopes, Resource partitioning, δ^13^C, δ^15^N, Trophic preferences

## Abstract

**Background:**

In sub-Saharan Africa, malaria, transmitted by the *Anopheles* mosquito, remains one of the foremost public health concerns. *Anopheles gambiae*, the primary malaria vector in sub-Saharan Africa, is typically associated with ephemeral, sunlit habitats; however, *An. gambiae* larvae often share these habitats with other anophelines along with other disease-transmitting and benign mosquito species. Resource limitations within habitats can constrain larval density and development, and this drives competitive interactions among and between species.

**Methods:**

We used naturally occurring stable isotope ratios of carbon and nitrogen to identify resource partitioning among co-occurring larval species in microcosms and natural habitats in western Kenya. We used two and three source mixing models to estimate resource utilization (i.e. bacteria, algae, organic matter) by larvae.

**Results:**

Laboratory experiments revealed larval δ^13^C and δ^15^N composition to reflect the food sources they were reared on. Resource partitioning was demonstrated between *An. gambiae* and *Culex quinquefasciatus* larvae sharing the same microcosms. Differences in larval δ^13^C and δ^15^N content was also evident in natural habitats, and *Anopheles* species were consistently more enriched in δ^13^C when compared to culicine larvae.

**Conclusions:**

These observations demonstrate inter-specific resource partitioning between *Cx. quinquefasciatus* and *An. gambiae* larvae in natural habitats in western Kenya. This information may be translated into opportunities for targeted larval control efforts by limiting specific larval food resources, or through bio-control utilizing competitors at the same trophic level.

## Background

Larval density and inter-/intra-specific competition have a significant effect on the development and growth rates of mosquito larvae in their aquatic habitats [1-3]. Higher larval densities and competition between *An. gambiae* s.s. and *An. arabiensis* result in extended development times, and several studies indicate that this may be a direct result of increased competition for microbial food resources [3-5]. This is particularly noteworthy for *An. gambiae* s.l*.* larvae, because they typically inhabit temporary, sunlit pools and increased development times may exceed the duration of aquatic habitats [6-8]. Although larval competition for food resources likely plays an important role in mosquito habitat productivity, relatively few studies have examined the actual partitioning of resources between and among species [9].

Extensive work has been carried out in characterizing larval habitats of malaria vectors. *An. gambiae* larvae have been found in a suite of habitat types including abandoned goldmines, brick making pits, ponds, agricultural drainage ditches and puddles [10,11]. When habitat size falls below ~1 m^3^, habitat stability and pupal occurrence decreases rapidly [11]; however, predation of larvae is typically lower and water temperatures are often higher in smaller habitats [12,13]. Minakawa found a significant correlation between habitat availability and the density of indoor resting mosquitoes in homes [6].

Stable isotopes represent a valuable yet not fully realized technology for mosquito ecology. Stable isotopes are convenient, naturally occurring tracers that are safe and non-radioactive. There are few examples of stable isotopes being used in vector biology studies; however, Hood-Nowotny and Knols illustrated a wide range of potential applications of stable isotopes in entomological studies and the falling costs and rapidly improving technology of stable isotope analysis [14]. Hood-Nowotny and others demonstrated the use of ^13^C enrichment as a population marker in a sterile insect technique context [15]. Recently, stable isotopes were used to identify the detrital resource base of *Aedes triseriatus* in tree holes [16]. Although natural abundance stable isotope studies have been used for several decades to clarify trophic relationships in aquatic ecosystems [17-19], few have specifically looked at resource partitioning among co-occurring species of mosquito larvae. Stable isotope ratios of carbon and nitrogen in invertebrate larvae reflect the larval diet [20], thus differences in larval diet should be reflected in larval isotope signatures. Winters and Yee were able to demonstrate a higher proportion of δ^15^N in *Aedes albopictus* relative to *Culex restuans,* which was indicative of a stronger association of *Ae. albopictus* larvae with detritus as a food resource [9].

Stable isotopes may also provide a means to examine the relationship between larvae and their potential predators. We have previously determined dragon fly nymphs to be one of the most efficient larval predators in the region and in microcosm studies, *An. gambiae* DNA was detected in dragonfly nymph guts [21].

In this study, we sought to use naturally occurring stable isotopes to examine inter- and intra-specific resource partitioning among larval mosquitoes. We examined inter- and intra-specific resource partitioning by measuring the carbon and nitrogen stable isotope ratios of larvae sharing the same habitats in western Kenya. We hypothesized that stable isotope ratios of carbon and nitrogen would be able to identify resource partitioning among larval species, and that *Anopheles* sp. would have isotopic signatures indicative of strong competition for resources, while differences in culicine and anopheline feeding strategies would demonstrate significant resource partitioning among the two genera. Dragon fly nymphs were also collected to identify stable isotopic signatures indicative of food choice. We also conducted a laboratory control study to assess trophic shift, or the difference in δ^13^C and δ^15^N composition of larvae compared to their food resources caused by larval metabolism [22]. Finally, to compliment our laboratory and field experiments, we conducted microcosm experiments to demonstrate resource partitioning between genera in relatively simple environments, lacking the complexity of natural habitats.

## Methods

### Laboratory experiments

*Anopheles gambiae* G3 strain mosquitoes, obtained from Malaria Research and Reference Reagent Resource Center (http://www.mr4.org), were used in the laboratory experiments. *Culex quinquefaciatus* larvae reared on Tetramin fish food (Spectrum Brands, Inc., Madison, WI, USA) were obtained from the Anthony James laboratory at UCI. Mosquitoes were reared in a walk-in insectary regulated at a relative humidity of 75%, temperature of 26°C, and 12:12 hour light–dark cycle. Mosquito larvae were reared in trays of 100 first-instar larvae per liter of water. *Cx. quinquefaciatus* and *An. gambiae* larvae were reared from first instar on fish food in order to determine the trophic shift associated with larval metabolism. This also served to identify differences in isotopic discrimination between the two species. *An. gambiae* larvae were reared on two different food types, fish food or brewer’s yeast, to test the utility of stable isotopes in determining what food resources were consumed. Third instar larvae were collected into paper cups and approximately 30 minutes were allowed for gut clearance before larvae were rinsed three times with distilled water. Larvae (1 mg/sample) and food samples (2.6 mg/sample) were oven dried overnight at 50°C prior to sample weighing and processing. Three samples were analyzed for each sample type, and for larval samples up to four larvae were pooled to obtain adequate sample weight for analysis.

### Microcosm experiments

To examine resource partitioning in a more controlled setting relative to natural habitats, we established an array of microcosms at the Kenya Medical Research Institute (KEMRI) in Kisian, Kenya. The microcosms were constructed using 60 cm diameter washtubs and approximately four liters of local top-soil were added to each. Soil was well mixed prior to addition to keep soil as similar as possible across microcosms. Rainwater was collected from roof runoff in Kisian, Kenya and approximately six liters were added to each microcosm. Microcosms were placed in full sun, screened with a light mesh to prevent oviposition by wild mosquitoes and left for five days to allow for microbial colonization prior to addition of larvae as done previously [23]. Colonies of *Cx. quinquefasciatus* and Kisian strain *An. gambiae* s.s. were used for microcosm experiments. The colonies were maintained at the insectary in Kisian. One hundred first instar larvae of each species were added to each microcosm. Water levels were maintained as needed by the addition of rainwater. Third and fourth instar larvae were collected, held for 30 minutes for gut clearance and rinsed three times with distilled water. Detritus and filamentous algae samples were also collected. Filamentous algae were rinsed thoroughly with distilled water prior to desiccation. Samples were dried for 48 hours in a desiccator, and stored with desiccant for transport to the United States further processing. Three replicates were taken for analysis for each sample type, and for larval samples desiccated larvae were ground and up to four larvae were pooled per sample to obtain adequate weight for analysis.

### Natural habitat sample collection

Sampling of natural habitats was conducted in Iguhu, Kenya (1,500 m above sea level) in the western Kenya highlands between October 2009 and August 2010. Four selected natural habitats consisted of abandoned goldmines and agricultural drainage ditches. Two habitat types were utilized in order to determine if the degree of resource partitioning was consistent between habitats. Habitats were chosen based on the co-occurrence of two or more mosquito species, at least one of which was *An. gambiae*. Sample collections were made from each habitat on a single day. Third and fourth instar larvae were collected using a standard dipper and transferred to the lab on ice in water from their respective habitats. Larvae were allowed several hours for gut clearance. Detritus and filamentous algae samples were collected and prepared using the same methods as the microcosm experiments. In 2011, allochthonous C3 plant material cow dung and dragon fly nymphs were collected in addition to larvae. Dragonfly nymphs were held for several hours to allow for gut clearance, and cleaned with distilled water. Samples were prepared for processing following the same methods as those in the microcosm experiments.

### Stable isotope analysis

Samples of insects (0.75 mg/sample), detritus (15 mg/sample), algae (1.5 mg/sample), and cow dung (1.5 mg/sample) were analyzed for ^13^C and ^15^ N isotopes at the University of California, Davis Stable Isotope Facility. Detritus samples were fumed for 12 hours with concentrated HCl to remove inorganic carbon. Samples were analyzed using a PDZ Europa ANCA-GSL elemental analyzer interfaced to a PDZ Europa 20–20 isotope ratio mass spectrometer (Sercon Ltd., Cheshire, UK). Samples were combusted at 1000°C in a reactor packed with chromium oxide and silvered cobaltous/cobaltic oxide. Following combustion, oxides were removed in a reduction reactor (reduced copper at 650°C). The helium carrier then flowed through a magnesium perchlorate water trap. N_2_ and CO_2_ were separated on a Carbosieve GC column (65°C, 65 mL/min) before entering the IRMS. During analysis, samples were interspersed with several replicates of at least two different laboratory standards. These laboratory standards, which were selected to be compositionally similar to the samples being analyzed, have been previously calibrated against NIST Standard Reference Materials (IAEA-N1, IAEA-N2, IAEA-N3, USGS-40, and USGS-41). Each sample’s preliminary isotope ratio was measured relative to the reference gases analyzed with each sample. These preliminary values were finalized by correcting the values for the entire batch based on the known values of the included laboratory standards, which results in corrected δ^13^C and δ^15^N values. δ^13^C values are typically depleted in the heavier isotope (negative) while δ^15^N values are enriched (positive) relative to international standards [14]. Samples from goldmine three and the microcosm experiments were obtained in 2011 and were analyzed using similar, previously published protocols at the University of California, Irvine Stable Isotope Facility [24].

### Data analyses

Data analyses were carried out using JMP Statistical Discovery Software statistics program (SAS Institute, Cary, NC). For laboratory and microcosm experiments, differences in δ^13^C and δ^15^N between *Cx. quinquefaciatus* and *An. gambiae* were compared using the paired student’s t-test. Welch’s t-test was used to compare δ^13^C and δ^15^N of mosquito species with samples that had unequal variance. Separate tests were used for ^13^C and ^15^N. We used analysis of variance (ANOVA) to compare δ^13^C and δ^15^N signatures of the co-occurring mosquito species within each habitat for the natural habitat experiments. To examine the relative contribution of potential food sources to target species, we used two and three source mixing models made available by Phillips and Gregg (IsoError, version 1.04; http://www.epa.gov/wed/pages/models/stableIsotopes/isotopes.htm) [25]. Two and three source mixing models allow for estimation of the proportional contributions of food sources to the consumer, and they take into account the mean, standard deviations and sample number for both δ^13^C and δ^15^N values.

## Results

### Laboratory experiments

**Figure 1:**
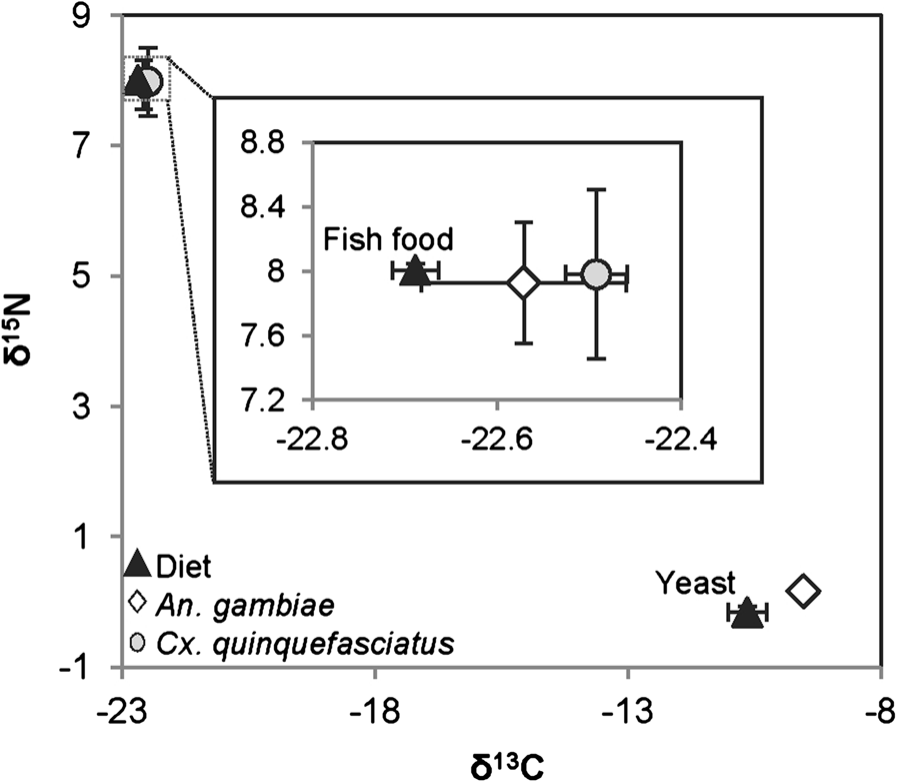
**δ**^**13**^**C and δ**^**15**^**N composition of laboratory reared *Anopheles gambiae *and *Culex quinquefasciatus *on fish food and *An. gambiae *reared entirely on yeast.** The inset panel expands the cluster showing isotopic ratios of *An. gambiae*, *Cx. quinquefasciatus* and their laboratory diet (fish food).

We conducted experiments in the laboratory to determine whether carbon and nitrogen stable isotope ratios were able to indicate food resource differences and similarities between *Cx. quinquefasciatus* and *An. gambiae* larvae. Carbon and nitrogen isotopic signatures of *Cx. quinquefasciatus* and *An. gambiae* larvae reared from first instar on Tetramin fish food were not significantly different (δ^13^C, t = 0.66, df = 4, P = 0.54; δ^15^N, t = 0.09, df = 4, P = 0.93) (Figure 1). Both larval species were slightly enriched in ^13^C when compared to the dietary source; however, the carbon enrichment was negligible relative to isotopic discrimination found in microcosm and natural habitat experiments. When *An. gambiae* first instar larvae from the same laboratory colony were reared on yeast or fish food alone, the carbon and nitrogen isotopic signatures reflected the food source (*An. gambiae* reared on fish food and yeast; Welch’s t-test; δ^13^C, t = 104.20, df = 3.0, P < 0.0001; δ^15^N, t = 19.97, df = 2.2, P = 0.0015) (Figure 1).

### Microcosm experiments

**Figure 2:**
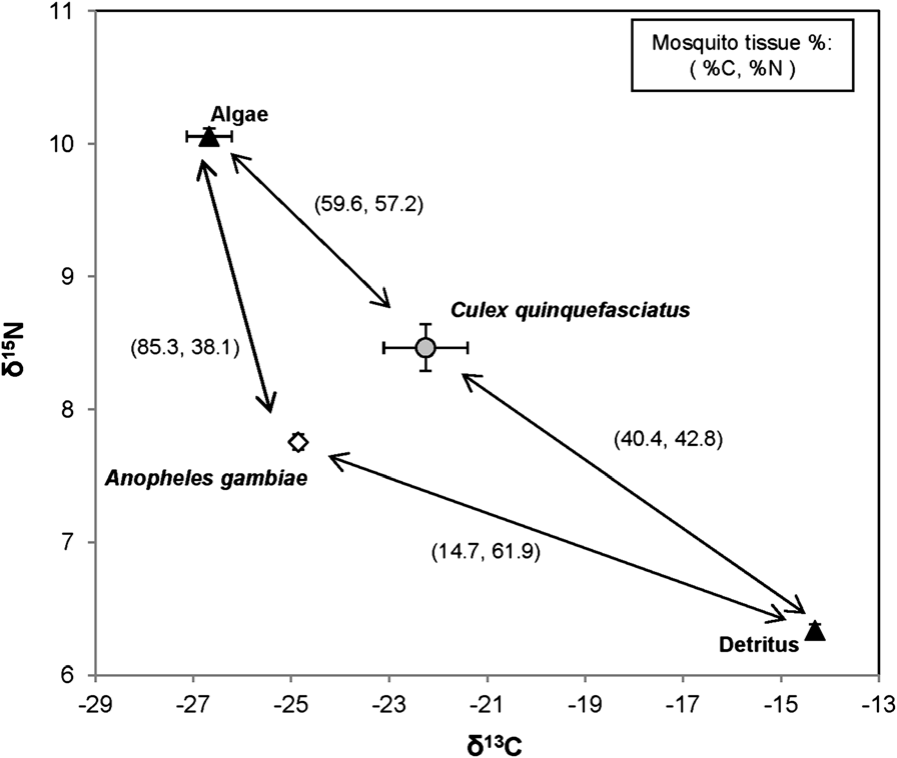
**Microcosm experiment δ**^**13**^**C and δ**^**15**^**N values for two larval species and two potential food resources.** Arrows connect larvae to their potential food resources, and numbers in parentheses are the estimated percent carbon and percent nitrogen (%C, %N) contribution.

In microcosms with equal proportions of *Cx. quinquefasciatus* and *An. gambiae* we demonstrated significant differences in both δ^13^C (t = 2.57, df = 5, *P* = 0.0499) and δ^15^N (t = 3.33, df = 5, *P* = 0.0209) larval body tissue (Figure 2). A two source mixing model suggested that detrital and algal food resources contributed to mosquito tissue of both species. Elemental analysis of *An. gambiae* body content showed that 85.3% of and 38.1% of body nitrogen was attributed to algal resources, compared to 59.6% and 57.2% in *Cx. quinquefasciatus*, respectively (Table 1). While *Cx. quinquefasciatus* appeared to forage about equally between the algal and detrital, the isotopic signatures for *An. gambiae* was skewed (enriched in ^15^ N and depleted in ^13^C), suggesting another, un-analyzed food source may have been contributing to the *An. gambiae* diet (Figure 2).

**Table 1:** **Two source mixing model estimates of dietary contributions to *An. gambiae *** **s.s. and *Cx. quinquefasciatus *** **larvae reared in microcosms**

**Mosquito tissue (est. % contribution) ± SE**				
	***An. gambiae***		***Cx. quinquefasciatus***	
	**Carbon**	**Nitrogen**	**Carbon**	**Nitrogen**
**Filamentous algae**	85.3 ± 1.6	38.1 ± 1.8	59.6 ± 0.6	57.2 ± 2.9
**Detritus**	14.7 ± 1.6	61.9 ± 1.8	40.4 ± 0.6	42.8 ± 2.9

### Natural habitat sample collections

**Figure 3:**
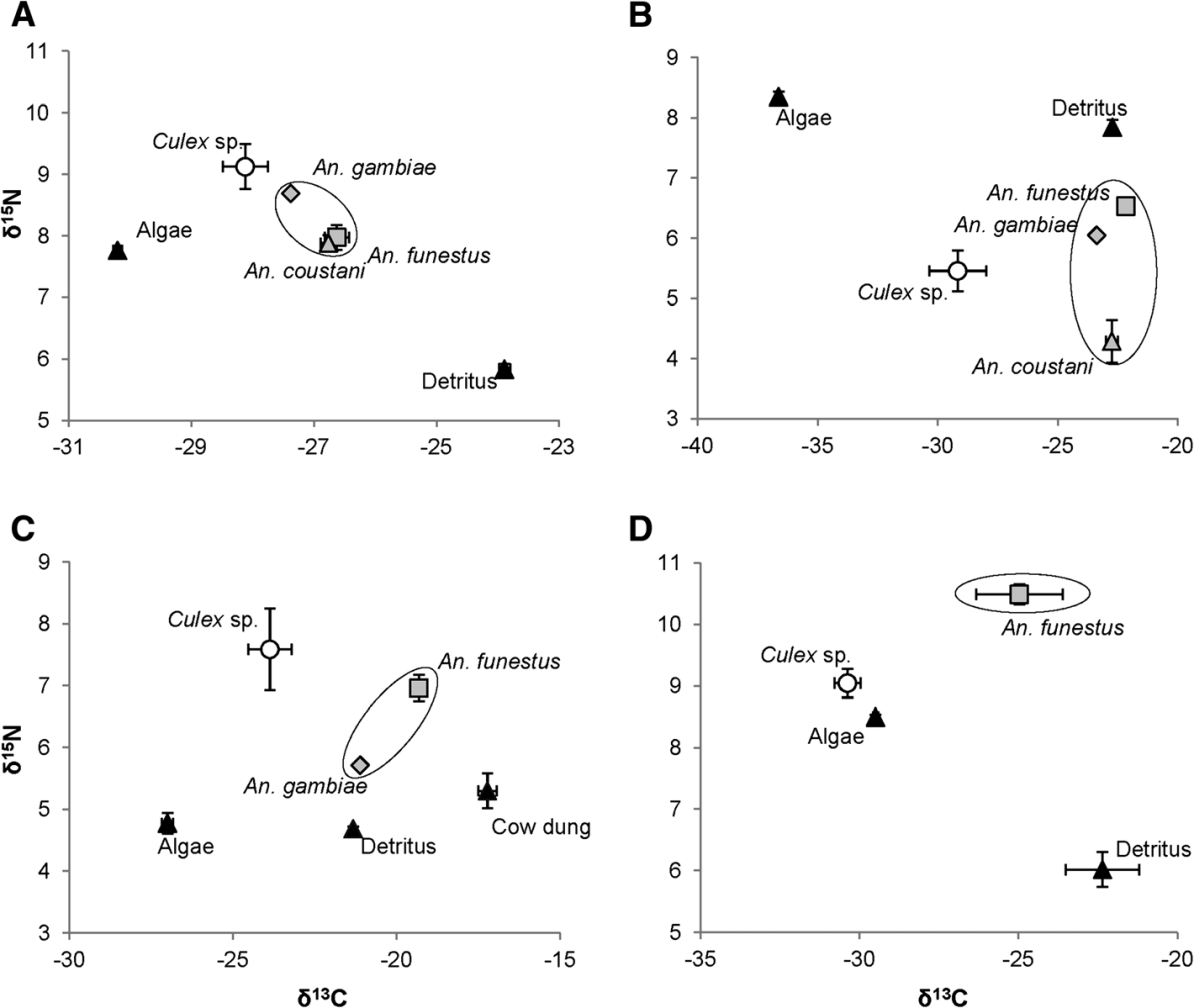
**δ**^**13**^**C and δ**^**15**^**N values for larval species and food sources collected from natural habitats.** Panels **A-D** show results from individual habitats that were sampled (Table 2). All larval species present in a habitat were sampled.

**Figure 4:**
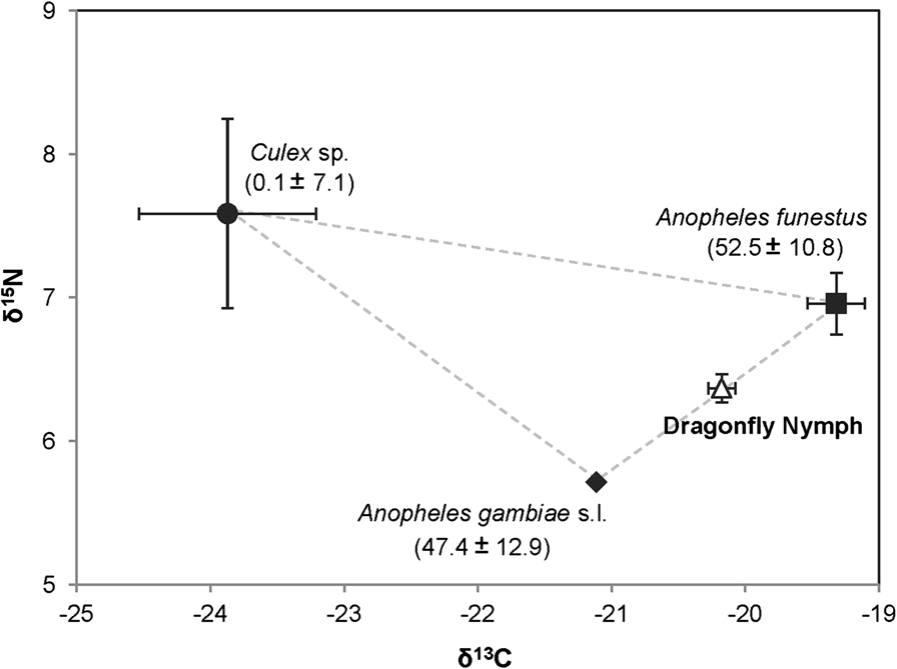
**Three source, two isotope mixing model.** Results estimate the dietary contribution (% ± SE) of *An. gambiae* and *Cx. quinquefasciatus* to dragonfly nymphs sampled from the same natural habitat.

In all four habitats *Culex* sp. were consistently depleted in δ^13^C relative to *An. gambiae*. However, δ^15^N did not clearly demonstrate partitioning of resources (Table 2). In all habitats with multiple anopheline species, the anophelines generally clustered together which may reflect the differences in morphology and foraging strategies between the two genera (Figure 3, Table 2). In 2011, we sampled cow dung from near the habitat edge as a third potential food source. A three source, dual isotope mixing model was not able to resolve the relationships of larvae to the potential food sources, suggesting the need for a more comprehensive sampling of potential food sources. Dragonfly nymphs were sampled from the same habitat. In the present study, a mixing model using isotopes of carbon and nitrogen suggested that *An. funestus* (53%) and *An. gambiae* (47%) make up the majority of contribution to dragonfly nymphs when compared to *Culex sp.* (0.1%), (Figure 4).

**Table 2:** **ANOVA results of co-occurring anopheline and culicine δ**^**13**^**C and δ**^**15**^**N signatures from natural habitats**

	**δ**^**13**^**C**		**δ**^**15**^**N**	
**Habitat**	**F**_**df, error**_	***P***	**F**_**df, error**_	***P***
**A) Goldmine 1**	5.07_1,7_	0.0591	10.91_1,7_	**0.0131**
**B) Goldmine 2**	48.82_1,6_	**0.0004**	0.23_1,6_	0.6460
**C) Goldmine 3**	29.65_1,6_	**0.0016**	2.98_1,6_	0.1353
**D) Drainage ditch**	14.54_1,4_	**0.0189**	25.70_1,4_	**0.0071**

Habitat letters refer to panels in Figure 3. Significant P values are in bold.

## Discussion

Laboratory experiments to determine the degree of trophic shift associated with larval metabolism demonstrated low levels of fractionation. Larvae of both *An. gambiae* and *Cx. quinquefasciatus* closely reflected their laboratory chow (fish food) diet. Further, when *An. gambiae* from the same colony were reared on brewer’s yeast, their isotopic composition was indicative of this alternative diet. In the subsequent field experiments, larval genera and species showed variable isotopic compositions consistent with laboratory results, which is indicative of resource partitioning. Kaufman also found that larval carbon and nitrogen isotopes reflected their food source [16]; however, higher fractionation in larvae was observed when compared to the current study. Stable isotope values of δ^15^N in *Ae. albopictus* and *Cx. restuans* have suggested differences in assimilation or utilization efficiency of nitrogen [9]. In our laboratory studies the low level of trophic shift associated with *An. gambiae* and *Cx. quinquefasciatus* feeding allowed us to directly interpret differences in larval isotopic composition as evidence of resource partitioning in the subsequent microcosm and natural habitat experiments.

The partitioning of resources in the microcosms was striking, as evidenced by the relative differences between larval *An. gambiae* and *Cx. quinquefasciatus* δ^13^C and δ^15^N signatures in microcosms. This is indicative of differential feeding on and/or assimilation of the resources available in microcosms. We hypothesized that partitioning would be less apparent in microcosms when compared to natural habitats. Bacteria, algae and protozoans make up a large proportion of the larval diet and can vary widely in different habitats depending on habitat duration, soil type and allochthonous inputs [26-28]. Due to limitations of food source diversity (i.e. little to no allochthonous plant material, debris, and a limited period for microbial growth), opportunities for larvae to forage on distinct resources are limited. Although the resources in microcosms are presumably less diverse than those found in natural habitats, we found more pronounced differences in microcosms. In addition to a highly diverse menu for larvae in natural habitats, difficulties in identifying resource partitioning in natural habitats using stable isotope analysis alone is also likely due to the long term mixing and nutrient turnover these habitats experience [14]. The resource portioning we found between these commonly co-occurring larvae may offer opportunities for species-specific targeted control. Shading habitats or using algaecides may have a negative impact on larvae associated with emergent algae and photosynthetic microbes, such as *An. gambiae*. For example, in western Kenya, shading agricultural drainage ditches with Napier grass, a regularly cultivated cow fodder, reduced larval populations of *An. gambiae* s.l. by 75-80% [29]. As observed, the shading did result in lower temperatures which can affect larval development [30]; however, it is likely that changes in the aquatic microbial community structure in response to shading may have played a role in the developmental success of larvae, and perhaps oviposition via volatile cues to the gravid female. Some of these interventions may not fully eliminate the target larval species, but in some cases, population reduction may lead to competitive exclusion of the target species.

Dragonfly nymphs had an isotopic composition indicative of a primarily anopheline diet. Although this result should be interpreted somewhat carefully since only three food sources were taken into account (*Cx. quinquefasciatus*, *An. gambiae* and *An. funestus*), studies suggest that culicine larvae may have a competitive advantage over anophelines when dragon fly nymphs are present in the habitat [2,31]. Our group reported a 70% reduction of *An. gambiae* s.s. larvae exposed to dragonfly nymphs, and used PCR to confirm the presence of *An. gambiae* DNA in the nymph gut content [21]. There may be other significant contributors to the nymph diet, but these resources would presumably occupy the same trophic level of the anophelines. Microcosm experiments with known predators and prey may help to evaluate the role of tropic structure in determining mosquito species success and habitat productivity.

In addition to more comprehensive food source sampling, stable isotopes may be useful in studying other aspects of vector ecology. Stable isotope ratios of oxygen and hydrogen are significantly affected by processes of evaporation and infiltration of ground water and rainwater, which will vary based on habitat size [14,32]. Thus, naturally occurring stable isotopes may potentially be used to address other problems in mosquito ecology, such as tracking adult dispersal from larval habitats. Variation in larval development conditions results in variable adult mosquito size and fitness [3,33], and determining the aquatic source of “successful” adult mosquitoes will provide useful insights for targeted vector control programs. Commercially available enriched compounds can also be used for such studies in the laboratory and the field. For example, Hamer *et al*. recently used ^15^ N-labeled potassium nitrate and ^13^C-labeled glucose to monitor dispersal of naturally breeding *Culex pipiens* mosquitoes [34].

As resource utilization and partitioning among larval mosquitoes can play a major role in determining mosquito species composition and survival in aquatic habitats, many studies have addressed the larval mosquito diet and identified differences in food preference largely based on gut content analyses [35,36]. The advantage of using a stable isotope method to examine resource partitioning is that carbon and nitrogen isotopes are assimilated into the larval body and contribute to the larval body mass whereas gut content analysis does not necessarily indicate assimilation.

## Conclusions

Understanding the trophic relationships in larval mosquito habitats has broad implications for malaria vector management. Habitats with co-occurring culicine and anopheline larvae are common throughout western Kenya, and competitive interactions among co-occurring mosquito larval species play a role in regulating adult populations [1,2]. The current study demonstrated a high degree of resource partitioning between *Cx. quinquefasciatus* and *An. gambiae* larvae. This was most evident in the microcosm studies conducted in Kenya. Further, resource partitioning among culicines and anophelines in natural habitats was also demonstrated. This information can be translated into potential opportunities for targeted larval control efforts by limiting specific larval food resources or through bio-control, utilizing competitors at the same trophic level. Given that culicine and anopheline larvae have been shown to partition the resources available in their habitats, it may be possible to target key food resources as an indirect method of species-specific malaria control.

## Ethics statement

No specific permits were required for the described field studies. These locations were not protected land, and the field studies did not involve endangered or protected species.

## Competing interests

All authors declare they have no competing interest in this study.

## Authors’ contributions

TMG, GY and AKG conceived and designed experiments. TMG, YAA and EJK implemented and supervised the study. TMG did data analysis and interpretation. TMG wrote the manuscript. TMG, EJK and GY revised the manuscript. All authors approved the final version for submission.
